# The Mental Philosophy of Fichte the Younger
**Contributions to Mental Philosophy*. By Immanuel Hermann Fichte. Translated and edited by J. D. Morell, A.M. London. 1860.


**Published:** 1860-04-01

**Authors:** 


### Art. IV.—THE MENTAL PHILOSOPHY OF FICHTE THE YOUNGER.*

Under the title of "Contributions to Mental Philosophy," we are indebted to Mr. Morell for a translation of "Zur Seelenfrage, eine Philosophische Confession," a little treatise that has recently proceeded from the pen of the younger Fichte. The translator introduces his work by a prefatory chapter, in which his reasons for undertaking the task are set forth in considerable detail; and, before proceeding to the text of the volume, we will endeavour to lay an abstract of these reasons before the reader.

After devoting two or three pages to a very brief account of the biography, studies, and early life of his author, Mr. Morell expresses a belief that the treatise he has selected is calculated, not only to throw some light upon the singular development of modern German speculation, and the point to which it is now tending; but, also, to conduce generally to the interests of psychology. In support of the latter position he refers to the present state of the science, and to efforts for its advancement that have proceeded exclusively upon either the dualistic or the materialistic principle; neither of which has hitherto offered any prospect of satisfactory results. In the school of Fichte there is shown a way of escape from this old alternative; and a system of spiritualistic psychology, based upon the most complete induction of facts.

"Another ground," writes Mr. Morell, "upon which it appeared to me desirable to circulate the present little work in our own country was, that it shows with such remarkable clearness the bearing which psychological inquiries have upon other important questions." As illustrations, the questions of a conscious immortality, of the bearing of psychology upon the theistic argument, especially in relation to the Divine Personality, and of the nature of the abnormal phenomena comprised under the word "*mediumship*," are separately advanced and considered.

* *Contributions to Mental Philosophy*. By Immanuel Hermann Fichte. Translated and edited by J. D. Morell, A.M. London. 1860.

With regard to the first of these questions, Mr. Morell compares the two extreme views respecting the nature of the soul as an intelligent principle: namely, that which regards it as a manifestation of the universal and absolute reason, individualized by connexion with a bodily organism; and that which regards it as consisting of a series of phenomena necessarily springing out of a given nervous organization. To the first view he objects that, if it were the truth, the severance of the connexion would destroy individuality, and return the soul to the infinite, as a wave is lost again in the ocean; and, to the second, that it cuts off the very possibility of a continuous mental life, and, by making a physical apparatus essential to the manifestation of mind, compromises irrecoverably the whole hope of immortality. At the conclusion of this argument, we find the following striking passage:—

"I know it will be said that the fact of immortality is made known by direct revelation, and that the omnipotence of the Deity is not to be limited by any notions of impossibility which we may entertain. To which I reply, that nothing is more trying and unfortunate for our mental peace than cases in which the dictates of revelation are opposed to the obvious indications of science. Our faith is not so strong that it can afford to disregard the intimations of science, even when they are adverse to it; or to neglect them when they are confirmatory. *Scientific* evidence will always prove stronger in the long run than *mere belief;* for, as we cannot admit truth to be at variance with itself, we must necessarily, in the long run, relinquish our hold of that side of a contradiction on which the grounds are most open to dispute. For myself, I must freely confess, that my own inward convictions of a conscious immortality have involuntarily grown dim or vivid, almost exactly in proportion to the strength with which I have found the dependence or independence of mind upon physical conditions to be confirmed by scientific considerations. When the dependence indeed is made absolute, I cannot conceive that any mind much accustomed to logical consecutiveness can hold the doctrine of a life hereafter with any real tenacity." (p. xx.)

Upon the relations of psychology to the theistic argument, Mr. Morell makes no remarks, but passes on to consider how the principles defined by Fichte may be brought to bear upon the explanation of certain *abnormal phenomena* of the human mind, or in other words, the phenomena of "*mediumship.*" To this question it will be necessary to return hereafter.

Finally, we learn from the preface that Mr. Morell is himself engaged in an endeavour to construct a coherent and harmonious whole from the materials prepared by various labourers in the field of psychology, and "at least to *commence* the work of building up the science upon a broader and deeper foundation than has been usually attempted in our own country." He therefore sends forth this little book in order "that the thoughts of many may be directed to those questions which I hope in due time to discuss more fully, and as a kind of pioneer in the pathway of popular interest." Especially when regarded from this point of view, we cannot but regard the contributions to mental philosophy as a most seasonable and interesting publication, and as an earnest of a pledge which Mr. Morell will, we trust, speedily be enabled to redeem.

Passing on now from the translator's preface to the work itself, we find this cast in a mould of eclecticism that renders any attempt to abstract its contents extremely difficult and unpromising. The author has chosen the form of a free and personal expression of convictions in order that he may offer to the world a succinct programme of opinions, and give a general view of the grounds on which they are supported, without finding it necessary to adduce logical proof of every position that is maintained, or even to do more than hint at the nature of the evidence in the case. Hence (the treatise being a model of brevity and conciseness) there is scarcely a passage that could be omitted without injury to the general argument, or that could be condensed without injury to itself; and we are compelled to state the leading characteristics of the volume in the very words presented to us by the translator. In a chapter of "Introductory Remarks" the aim of the author is expressed as follows:—

"To bring out the fundamental idea of the nature of the soul from all the surroundings in which a complicated and critical inquiry necessarily envelopes it, and to state it anew upon its prominent and most decisive grounds, this will be my first and foremost endeavour. Having done this, it will become possible to cast a glance over the whole system of truth to which this idea belongs, and through which alone it can assume a deep meaning and a lasting value. Let us attempt, then, to express in a few simple words what was before laid down in the more complicated form of a scientific treatise.

"The human mind does not only possess *à priori* elements (primitive notions, primitive feelings, primitive efforts) in its consciousness, but it *is* in its own peculiar nature and composition, an *à priori* existence, *i.e.*, one whose character is impressed upon it *anterior to experience.*

"This is not intended to affirm that mind exists originally in the form of a mere impersonal Pneuma, or of abstract universal reason, as Hegel imagined it; for independently of the special psychological difficulties of this view, observation does not give us the very smallest intimation of any such uniform mental constitution in the fully developed man, but rather of the exact contrary—the most marked individuality. So far from that, we must regard the human mind as being, even in its primitive, pre-existent root, an individualized nature, a germ of personality, since the result of its actual life shows it really to be so; for it were a contradiction to suppose that individuality is added *on* to it from without, or that it is the mere fortuitous product of its connexion with external circumstances. This idea we have had to make good throughout all our discussions on particular questions. And if, at length, we found it necessary to attribute to the mind a kind of pre-existence anterior to its own *conscious* life, the question naturally arose respecting the nature of such pre-existence, and the general analogy by which it could be confirmed.

"And here the universal analogies of nature did not fail us. As certain as it remains impossible to deduce the higher steps of existence in nature (those included in animal and vegetable life) from the mere development of inorganic materials and processes; and yet as certain as the more perfect species of plants and the higher animals are the latest, and man the latest of all (while yet it is equally impossible that we should attempt to explain the higher animal, or man, by means of a gradual process of development from the lower); as certain, in a word, as every species of plant and animal must be regarded as having its own commencement and its own ground of explanation, we are constrained to form, in relation to the whole range of natural science, a universal idea of *pre-existence*, of which the pre-existence of the human mind is only a particular expression and an individualized result. Every distinct or individualized existence in nature (such as the species of plants and animals in the region of organic existence, and the individual mind of man in the region of spiritual being) must have eternally pre-existed, if it is possible that it should realize its individuality in time; for none of these individualities can be regarded as being indifferently of one stamp or another, just as we please, or as only having a temporary and fortuitous origin; but each in its kind is an integral part of a united whole, and must have been eternally planned in relation to the *particular* as well as the *universal* harmony of the universe.

"Accordingly, we find that we are constrained to admit the incontestable notion of pre-existence into the region of psychology, and to co-ordinate it with those analogies of nature to which the geological history of the earth conducts us. Here also already exist, potentially, the future species of vegetable and animal life; and that too in their entire individuality; for this it is which gives them their unalienable place in the eternal plan of the world. They acquire, however, *temporal* existence only when and so long as the *material* of life and the outward conditions of its realization meet together (in the process of the world's epochs) with the original type. Just so it is with the human monad; it requires the organic process of incorporation, in order for it to become endowed with consciousness. As soon, however, as the material of life is afforded it, the whole process of realization in time begins, first in the form of *incorporation*, and then of *consciousness*. In all this, be it observed, it is simply the original individuality of the mind which is developed, and comes to itself; inasmuch as that only can be unfolded in time which is prefigured in the eternal unity and place of all things." (pp. 2-5.)

The leading idea of the passage we have extracted is expounded and illustrated in several chapters, the first of which is devoted to considering the essential nature of the human soul. The argument is made to proceed from the pre-conscious to the conscious condition, upon the ground that the real or actual consciousness is based upon a potential one, *i. e.*, upon a middle condition of the soul, in which it already possesses the specific character of objective intelligence, but without being conscious of it. To such a condition the author attributes all instinctive operations, the art-instincts of insects, for example, rejecting the commonly-received opinion that these are due to physical laws impressed upon the nervous system by the Creator. He concludes that—

"*No organic activity is possible without the co-operation of thought, which thought unquestionably can only exist in the soul; inasmuch, however, as it precedes sensation* (*the principle by which consciousness is awakened*), *it must necessarily remain unconscious.*" (p. 19.)

It follows that not only the formative or creative ideas of the fancy, and the formative or creative acts performed by the perfect organism, are attributed by Fichte to the soul, but also the modelling and formation of the organism itself from its earliest rudiments. He regards the soul as the plastic power by which its temporary and material house, the human frame, is built up and put together; and each individual soul as being, so to speak, an individual providence for its own body during the continuance of the union between them. To put this view into somewhat different words, we might say that Fichte regards a soul as the medium of the Divine operation upon matter; an operation which we see exerted only under certain limitations imposed by physical laws. The soul

"— Has neither power to produce the real substantial elements, nor to draw them near by any force of dynamical attraction, nor to effect any change in their *quality*. In a word, the creation of matter and the change of matter, the *chemistry* of all the processes (though a necessary condition to all organic life), is wholly foreign to mental influences. This rests upon general and independent laws, under the conditions of which, indeed, the morphological activity is brought to a conclusion, but which it is not able in any way to modify or change. The soul is the *form*, and, at the same time, the formative principle of its body—its real prototype; but it can only realize itself by co-operation with a world possessing distinct elements of its own, and following distinct laws. Here accordingly, we can freely acknowledge with Lotze, a physico-psychical mechanism, that is, the subjection of the soul, in all its organic and conscious operations to a mechanism not explicable out of itself. The *non-ego*, to use an old phrase, is met by the *ego*, the soul, as a second, real, and independent power. This power, indeed, the soul can subdue and use for its own purposes, but only under certain definite limitations. This point in the whole system of arrangements must not be overlooked, for the traces of it are only too *visible*. The organism not only furnishes the soul with the necessary conditions of consciousness, it binds and limits also the power of consciousness itself; for in the freer states of the soul we can clearly trace the effect of the temporary suspension of these limitations." (p. 38.)

The conclusions of the author upon this subject (the essential nature of the human soul) may perhaps be summed up by saying that he regards it as an individual and distinctly personal *à priori* substance, possessing certain inherent powers which require to be developed and brought into action, or at least into the domain of consciousness, by the aid of a material organism of which the soul itself is both the plastic or formative agent, and also the basis of all individuality. The whole of the pre-conscious state of the soul is held to be essentially and specially a process of thinking; without, however, its thought as yet touching the threshold of consciousness.

The suggestion thrown out almost casually in the course of argument, and not again taken up or followed to its results, the suggestion, namely, that all organization is the work and proper evidence of a soul, *i. e.*, an individual soul other than the Divine Mind pervading all things, will appear very startling to most readers. In this country we are much accustomed to rest upon the notion that organic acts performed by the lower forms of animal life (the cited art-instincts, for example) are evidences only of "laws written upon the nervous pulp" by the finger of God. Still more are we accustomed to attribute vegetative life to the *direct* agency of the Creator; and the idea of souls pervading the inferior domains of existence is one that opens up an entirely new future to psychology. If this idea should hereafter be found to rest upon arguments that are unassailable, will it not probably be developed into a belief that the perfect self-consciousness of the soul is a state gradually brought about by connexion with a successively ascending scale of physical organisms. The very suggestion that such may be the case appears to throw some light upon the great moral problem that is involved in the daily misery brought upon the lower animals by man's sin; while, on the other hand, it seems a contradiction in terms to speak of a soul that shall be other than immortal, or to imagine immortality for the *individual* plastic principle of a plant or an insect.

The next chapter, under the title of "Primitive Consciousness and Sense Consciousness," is devoted to a glance at the inferences to be drawn from all the abnormal phenomena of the human mind, such as dreaming, visions, presentiments, clairvoyance, mediumship.

The author sets out with the postulate that there are a sufficient number of facts of this class, apart from all wilful or unconscious deception, to require explanation at the hands of the psychologist; and further, that the explanation commonly given is not exhaustive of the phenomena. Neither a chance coincidence between the products of the ordinary laws of association and the actual occurrences, nor a logical calculation of probabilities, the premises of which are *tacitly* present in the depths of the consciousness, is sufficient to account for all the cases which occur.

His general explanation of the class of facts referred to is based upon the belief that there exists, in every human mind, a higher region of thought than that which is reached through the medium of the senses, an *à priori* life in the midst of its empirical and conscious life. "Dreaming," he writes, "turns the inner side of the mind, which is ordinarily concealed, to the light of consciousness, and thus it is the only form in which the *other half* of its being, the background of its waking life, can be imaged forth even in transient flashes." It follows that he regards these flashes as evidences of preconscious being, and considers no system of psychology complete which does not recognise and include them; more especially such of them as transcend the ordinary possibilities of sensuous life. As an illustration of this class he takes an instance of clairvoyance, or a case in which a future or a distant event, one thoroughly fortuitous, incapable of being guessed on the principle of probability, is minutely and distinctly pictured to the mind in second sight.* Such a case as this, we read,

"— Is of extraordinary significance. The precise truth and perceptive reality of vision, even down to its smallest details, is on the one side the characteristic, on the other side the enigmatical element in it, which peculiarly needs explanation. In dream waking of the kinds before mentioned, it was possible to explain all that was characteristic in them from internal conditions springing out of the preconscious but special nature of the soul. This possibility now ceases; a prevision so peculiar, and entering so much into detail, cannot possibly spring from the preconscious region. It necessitates us to draw the astounding but unavoidable conclusion, that a real and perceptive

* The writer of this article, some years ago, called upon a widow lady, whose only son was then in New Zealand. The writer was received by the lady's daughter, who stated that her mother was too unwell to see visitors, having been much distressed during the previous night by a very painful dream. She dreamed that she saw her son pursued, struck down, and killed by two New Zealanders, whose countenances were pictured to her with perfect distinctness; and she related her son's dying exclamation. In due course the mails from New Zealand brought intelligence that verified this prevision in a general way. The young man was last seen by his companions flying for his life from two of the natives, who were believed to have killed him immediately afterwards, and that at the very time of his mother's dream. In this case, neither the faces of the murderers, nor the last words of the victim, could be compared with the details of the vision; but the general coincidence was remarkable; and the writer relates it because the dream was brought under his notice so long before its verification was received. He is able to state, moreover, from personal knowledge, that the lady was not one of those habitual dreamers who are almost certain to meet with a coincidence in the course of a lifetime.

knowledge lies at the basis, which consequently can have its seat only in the consciousness of a personal mind, and from this mind be carried over into the consciousness of the seer.

"Herewith we have a series of further consequences opened up, which carries us into a wholly unsuspected region, and one which has hardly been touched upon hitherto, still less considered from a scientific point of view. All that we have described is only possible under the supposition of the *immediate* influence of one mind upon another; and this would further necessitate us to admit a hidden fellowship of souls, underlying our ordinary consciousness and our daily communication through the senses.

"It must be admitted, in reference to this theory, that the general premises we have laid down in relation to the nature of the soul do not present any grounds against its possibility. If it is shown that the largest and most essential part of our mind is distinct from, and unexhausted by, our sensational experience, it can hardly be supposed that this element stands alone, apart from all relations, and without any influence beyond its own invisible region. Such a supposition were in the highest degree improbable. As our mind has its root beyond the world of sense, so will it stand, in a hidden and unsensuous way, in mutual communication with the real existences of this higher region, and that, too, with those who like itself hold intercourse with the world of sense, as also with those who are already removed from it.

"It need scarcely be remarked how unexpected a light spreads itself, upon this supposition, over emotions and relations in the human mind, which no one has been yet able expressly to deny, but for which no rational ground of explanation has been yet discovered. Here, I believe such an explanation has been found, and in such wise that no doubt can be thrown either on the reality of the general foundation, nor any limit set to the speciality of the facts. On the contrary, observation is directly appealed to, and it is demanded of observation that it should search into the extent and the depths of what here becomes possible. For here, in fact, the richest gradation of phenomena shows itself from the special prevision of worthless events down to the warning and prophetic voice of a Socratic *Daimon*, or to the most powerful and penetrating revelation of historical significance.

"We must here, however, draw a warning limitation. It would be altogether unjustifiable and arbitrary, in the case of all such visions, to imagine that they are direct communications from the Spirit of God himself. We cannot deny, on our side, that we discover in this the germ of a most destructive enthusiasm. The supposition of the agency of mind of higher order than what is now to be found in the human consciousness is all that is necessary. The fact that such a mind knows the future beforehand, to an extent beyond what it is granted *us* to know, nay, to foresee what to us is accidental, does not at all militate against its possessing a finite nature, nor transform it into a being incapable of ignorance and deception. That such a being may gaze over a higher region of casualties than we do, is *possible;* for what *we* deem fortuitous is really only that whose causal connexion escapes our vision, whether it may have its ground in the inextricable web of outward events, or in the hidden motives of human character. Chance, in fact, is only *appearance* (a relatively necessary appearance, it is true), which therefore may be dissipated by a more widely embracing view of the universe and its relations." (pp. 59-61.)

The fourth chapter is devoted to the "Organic double life of the Soul," and is occupied chiefly in stating and elucidating the idea that the nervous apparatus has a very appreciable effect in retarding and limiting the psychical operations. Upon this view, the abnormal phenomena of ecstasy, prevision, and the like, are supposed to depend upon a temporary loosening of the ordinary bonds which fasten down mind to matter; and to foreshadow the great increase of power that awaits the disembodied spirit.

The fifth chapter deals with "The Question of Method;" *i.e.*, with the nature and limitations of the inquiry, and it hardly admits of any condensation. The sixth has for its subject, "The psychological origin of our perceptions of Space;" and the author's views thereupon are illustrated at greater length than accords with the general brevity of the treatise. In explanation of this, we learn from Mr. Morell that the chapter is not that of the original, which is indeed wholly omitted, an article more recently written by Fichte, in his "Philosophical Journal," being inserted in its place.

The author in this article starts forth from the Kantian principle of the original existence of space-perception in our consciousness; and adds to it that this space-perception has its psychological origin in an original *feeling* of extension, which is inseparable from the consciousness of our own existence. He argues that the mind can only be endowed with this original feeling on the ground of its being, *ab initio,* a space-creating being; and deduces from the argument a confirmation of his belief that the soul is an *extended substance.*

The next, and concluding chapter, is entitled "General Retrospect and Prospect," and may briefly be described as a series of suggestions, showing the bearing of the author's philosophy upon the great problems of existence. The nature of the Providential operations, and the relations subsisting between the Deity and mankind, are hinted at, rather than discussed, everywhere in a spirit of piety and reverence, but with a full appreciation of the assistance which philosophy may afford to faith.

In thus noticing this little book, it has been our endeavour to place before the reader a general view of its contents, and, at the same time, wholly to abstain from criticism. We regard it as being suggestive throughout, rather than argumentative; and, as the author's grounds of conviction are nowhere fully stated, his results can hardly be assailed at present, even by those who may refuse assent to them. We shall look forward, with the most lively interest, to the more detailed evidence and reasoning which the present work leads us to expect; and, in the mean time, must use the "philosophical confession" chiefly as a new hypothetical standard, against which to measure and compare our old opinions. Its appearance in an English dress must be considered, we think, as adding largely to the debt of gratitude already due from the public to the distinguished translator.

---

### Art. V.—PINEL: A BIOGRAPHICAL STUDY.

